# A Metal‐Free Carbon Monoxide Prodrug Suppresses Metastasis of Pancreatic and Breast Cancer

**DOI:** 10.1002/advs.202519898

**Published:** 2026-03-20

**Authors:** Tiantian Zhang, Xiang Chen, Xiaoxiao Yang, Muskan Gori, Rohit Kumar Varshnaya, Dongning Liu, Cheryl Zhang, Michelle Yi Qin Chen, Zhengming Chen, George Zhang, Erika Patel, Qiyue Mao, Tan A. Ince, Chalet Tan, Binghe Wang, Yi‐Chieh Nancy Du

**Affiliations:** ^1^ Department of Pathology and Laboratory Medicine Weill Cornell Medicine New York New York USA; ^2^ Department of Chemistry Center for Diagnostics and Therapeutics Georgia State University Atlanta Georgia USA; ^3^ Biostatistics Division Department of Population Health Sciences Weill Cornell Medicine New York New York USA; ^4^ Department of Pharmaceutical Sciences University of Tennessee Health Science Center Memphis Tennessee USA

**Keywords:** breast cancer, carbon monoxide prodrugs, heme, HRG1, metastasis suppression, pancreatic cancer

## Abstract

Metastatic recurrence is the principal cause of cancer mortality. Pancreatic ductal adenocarcinoma (PDAC) and triple‐negative breast cancer (TNBC) recur frequently after apparently curative therapy. Carbon monoxide (CO) is an endogenously produced signaling molecule with cytoprotective properties, but clinical use of CO gas is constrained by safety and dose‐control challenges. Here, a metal‐free CO prodrug (**CO‐116**) suppresses metastatic progression in vivo. In experimental models of PDAC and TNBC, **CO‐116** reduces metastatic burden without evidence of overt toxicity while maintaining carboxyhemoglobin within physiological ranges. At an equivalent total weekly dose, dividing the dose into more frequent, lower administrations achieves greater efficacy than a single weekly dose, indicating schedule dependence. Mechanistically, **CO‐116** downregulates the heme importer HRG1 and attenuates a downstream CYP1B1–SP1 program; gain‐ and loss‐of‐function studies establish HRG1 as a functional mediator of metastatic progression and CO responsiveness. An independent cohort using a distinct metal‐free scaffold (**CO‐103**) likewise reduces metastasis in PDAC, supporting a scaffold‐independent class effect. These findings establish a mechanistically anchored, non‐inhaled CO strategy to suppress metastasis and motivate adjuvant development focused on schedule optimization and biomarker‐guided dosing.

## Graphical abstract

A metal‐free carbon monoxide (CO) prodrug, CO‐116, delivers controlled systemic CO without inhalation. CO suppresses the HRG1–heme axis, reducing intracellular heme availability. Attenuation of this pathway inhibits metastatic progression in pancreatic and triple‐negative breast cancer models.

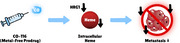

## 1 Introduction

Metastatic recurrence remains the principal cause of cancer mortality. Despite gains in public awareness and earlier detection, pancreatic ductal adenocarcinoma (PDAC) and triple‐negative breast cancer (TNBC) continue to show disproportionately high relapse rates after apparently curative therapy, underscoring the need for systemic strategies that safely suppress metastatic progression. In PDAC, up to 80% of patients who undergo surgical resection followed by chemotherapy experience disease recurrence [1, 2, 3]. Similarly, approximately 25% of patients with localized TNBC develop distant metastases, often within the first three years after diagnosis [4]. Once metastasis is established, current options rarely achieve durable control, and survival declines sharply. These realities highlight the need for nontraditional, low‐toxicity approaches for metastatic disease.

Carbon monoxide (CO), long viewed as a toxin at high doses, is also an endogenous molecule that contributes to organ homeostasis and exerts cytoprotective effects at low doses [5, 6, 7]. In healthy individuals, basal production yields ∼1%–2% carboxyhemoglobin (COHb) [8, 9]. Animal studies show therapeutic effects around 4%–10% COHb [10, 11], and carefully monitored human studies report tolerability at higher levels [9, 12]. Natural physiology offers precedent: deep‐diving mammals sustain higher endogenous CO (COHb ∼6%–10%), consistent with CO's anti‐inflammatory, cytoprotective actions [13, 14]. CO's exposure–response relationship and safety have been rigorously characterized, and clinical trials are evaluating low‐dose CO for organ transplantation, respiratory, and cardiovascular indications [5, 9, 11]. Physiological concentration of CO is higher than that of phenylalanine [9]. Studies in elite athletes have also explored CO exposure for performance benefits with COHb up to ∼10% under supervision [11, 12].

Collectively, these data support low‐dose, exposure‐controlled CO as a feasible therapeutic modality.

Despite this promise, the clinical use of inhaled CO gas is limited by safety and operational constraints. CO is colorless, odorless, and tasteless, making inadvertent exposure difficult to detect for patients and healthcare personnel, and precise dose control with gas is inherently challenging. To address these limitations, alternative delivery systems have been explored, including metal–carbonyl complexes (CORMs) [15], CO in solution [16, 17, 18], foam formulations [19, 20], and ultrasound‐responsive micelles [21]. However, growing evidence indicates that some metal‐based CORMs can cause metal‐associated toxicity and elicit CO‐independent effects [22, 23, 24, 25, 26, 27, 28, 29, 30, 31, 32, 33, 34]. In 2014, we reported the first non‐inhaled, metal‐free CO prodrugs [35], and since then, both our group [25, 29, 30, 36, 37, 38, 39, 40, 41, 42, 43] and the Larsen lab [44, 45] have developed metal‐free CO prodrugs that extrude CO via cheletropic loss from norbornadienone scaffolds. Leveraging Diels–Alder chemistry, we constructed a structurally diverse family of metal‐free organic CO prodrugs to enable safer, controlled, non‐inhaled CO delivery. This design avoids the logistical hazards of gas administration and the potential metal‐associated liabilities of some classical donors, while enabling therapeutic exposure control.

Beyond its established cytoprotective and anti‐inflammatory roles, low‐dose CO has emerging relevance in cancer biology. In preclinical settings, inhaled low‐dose CO inhibited primary cancer growth in the mouse models of prostate cancer, lung cancer, and pancreatic cancer [46, 47]. Previous work from our group demonstrated that inhaled low‐dose CO reduces cancer cell migration and suppresses metastatic outgrowth and post‐resection recurrence, improving survival in breast and pancreatic models [48]. Mechanistically, we demonstrated that low‐dose inhaled CO suppresses cancer cell migration by downregulating the heme importer HRG1, leading to reduced intracellular heme availability and attenuation of a downstream CYP1B1–SP1 transcriptional program [48]. While these findings implicated HRG1‐dependent heme metabolism as a CO‐responsive pathway, the functional relevance of this axis to metastatic progression in vivo, and its responsiveness to a non‐inhaled CO prodrug, remained unknown.

Motivated by these observations and by the practical limitations of gaseous CO, we asked whether a non‐inhaled CO prodrug could suppress metastasis in vivo and sought a mechanistic framework suitable for translation. Here, we show that **CO‐116**, a metal‐free CO prodrug, suppresses metastatic outgrowth in PDAC and TNBC models with acceptable tolerability and physiological systemic exposure. A schedule comparison reveals the superiority of more frequent, lower dosing over a single higher weekly dose at equal cumulative exposure. At the mechanistic level, **CO‐116** downregulates the heme importer HRG1 and attenuates a downstream CYP1B1–SP1 program; gain‐ and loss‐of‐function studies identify HRG1 as a functional mediator of metastatic progression and of the response to CO‐based interventions. To our knowledge, these results constitute the first demonstration that a carbon monoxide prodrug can inhibit metastasis in vivo and provide a rationale for developing non‐inhaled CO therapeutics to prevent recurrence and metastasis while preserving quality of life.

## 2 Results

### 2.1 CO‐116 Suppresses Liver Metastasis in an Experimental PDAC Model

**FIGURE 1:**
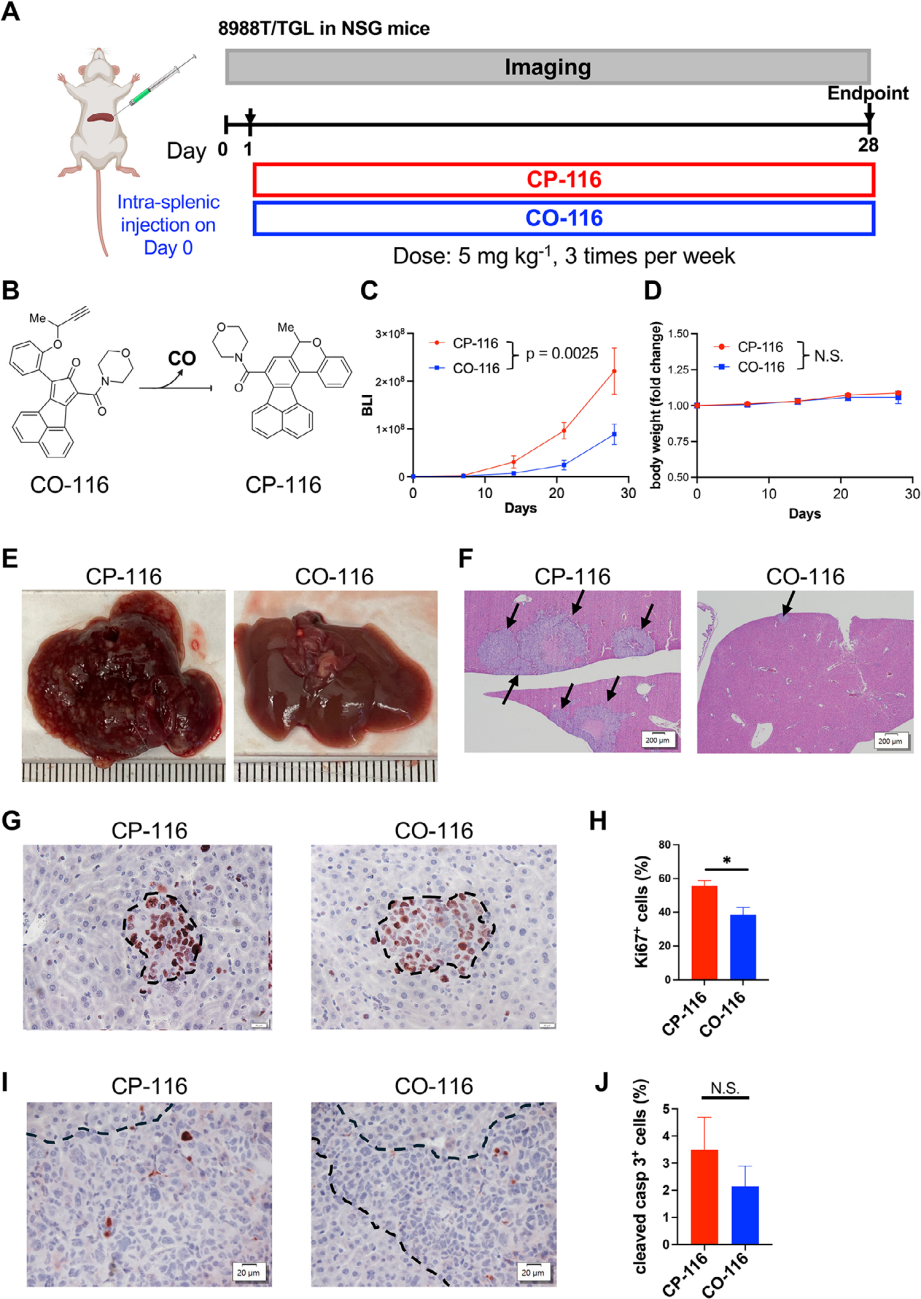
CO‐116 suppresses liver metastasis in an experimental PDAC metastasis model in part by reducing tumor cell proliferation. (A) Schematic of the experimental design. NSG mice were intrasplenically injected with 8988T/TGL PDAC cells. One day later, mice were randomized into two groups (*n* = 6 per group): **CO‐116** (5 mg kg^−1^, IV, three times per week) or **CP‐116** control (5 mg kg^−1^, IV, three times per week). (B) Structures of **CO‐116** and **CP‐116** and the chemical reaction. (C) Quantification of bioluminescence signals over time. Data are presented as mean ± SEM. Statistical significance was determined using GEE to account for repeated measurements over time. *p* = 0.0025. (D) Longitudinal fold change in body weight. Statistical significance was determined using GEE to account for repeated measurements over time. N.S.: Not significant. (E) Representative liver images at necropsy. (F) Representative images of H&E‐stained liver sections. Arrow: metastasis. Scale bar: 200 µm. (G) Representative images of Ki67‐stained liver sections. (H) Quantification of proliferative index in the metastases. Data are presented as mean ± SEM. Statistical significance was determined using a *t*‐test. ^*^ *p* ≤ 0.05. Scale bar: 20 µm. (I) Representative images of cleaved caspase 3‐stained liver sections. (J) Quantification of apoptotic index in the metastases. Data are presented as mean ± SEM. Statistical significance was determined using a *t*‐test. N.S.: Not significant. Scale bar: 20 µm.

To test whether a metal‐free CO prodrug restrains PDAC metastasis, we used an intrasplenic liver metastasis model with 8988T/TGL cells (Figure 1A). One day after tumor inoculation, NSG mice were randomized to **CO‐116** (5 mg kg^−1^, IV, three times per week) or **CP‐116** (the side product after CO release) control (5 mg kg^−1^, IV, three times per week). **CO‐116** is a metal‐free CO prodrug engineered for controlled, safe delivery of CO upon contact with aqueous environments [36, 49, 50]. Upon administration, **CO‐116** undergoes a hydrophobicity‐driven Diels–Alder reaction to release CO (Figure 1B). Longitudinal bioluminescence imaging (BLI) showed a significant reduction in metastatic burden in the **CO‐116** group (Figure 1C; Δslope = −0.039 log day^−1^, *p* = 0.0025, Generalized Estimating Equations (GEE)). No treatment‐related morbidity was observed, and body weight trajectories were comparable between groups (Figure 1D, *p* = 0.4177). No overt behavioral abnormalities (e.g., changes in activity, grooming, posture, or mobility) were observed in either group during routine daily monitoring. At necropsy (28 days post‐tumor injection), macro‐metastases were present in all **CP‐116** controls but absent in **CO‐116**–treated mice (Figure 1E). Histology confirmed significantly fewer and smaller hepatic foci with **CO‐116** (Figure 1F). Immunohistochemistry (IHC) demonstrated reduced Ki67 (Figure 1G,H) without a difference in cleaved caspase‐3 (Figure 1I,J), indicating decreased proliferation rather than increased apoptosis.

Independent of CO‐116, a different scaffold CO prodrug, **CO‐103** [51], administered in the same PDAC model, similarly reduced hepatic metastatic burden versus its matched **CP‐103** control (Figure S1), supporting the anti‐metastasis effect of metal‐free CO prodrugs.

### 2.2 Dosing Schedule: Frequent Low Dose Outperforms Once‐Weekly High Dose

**FIGURE 2:**
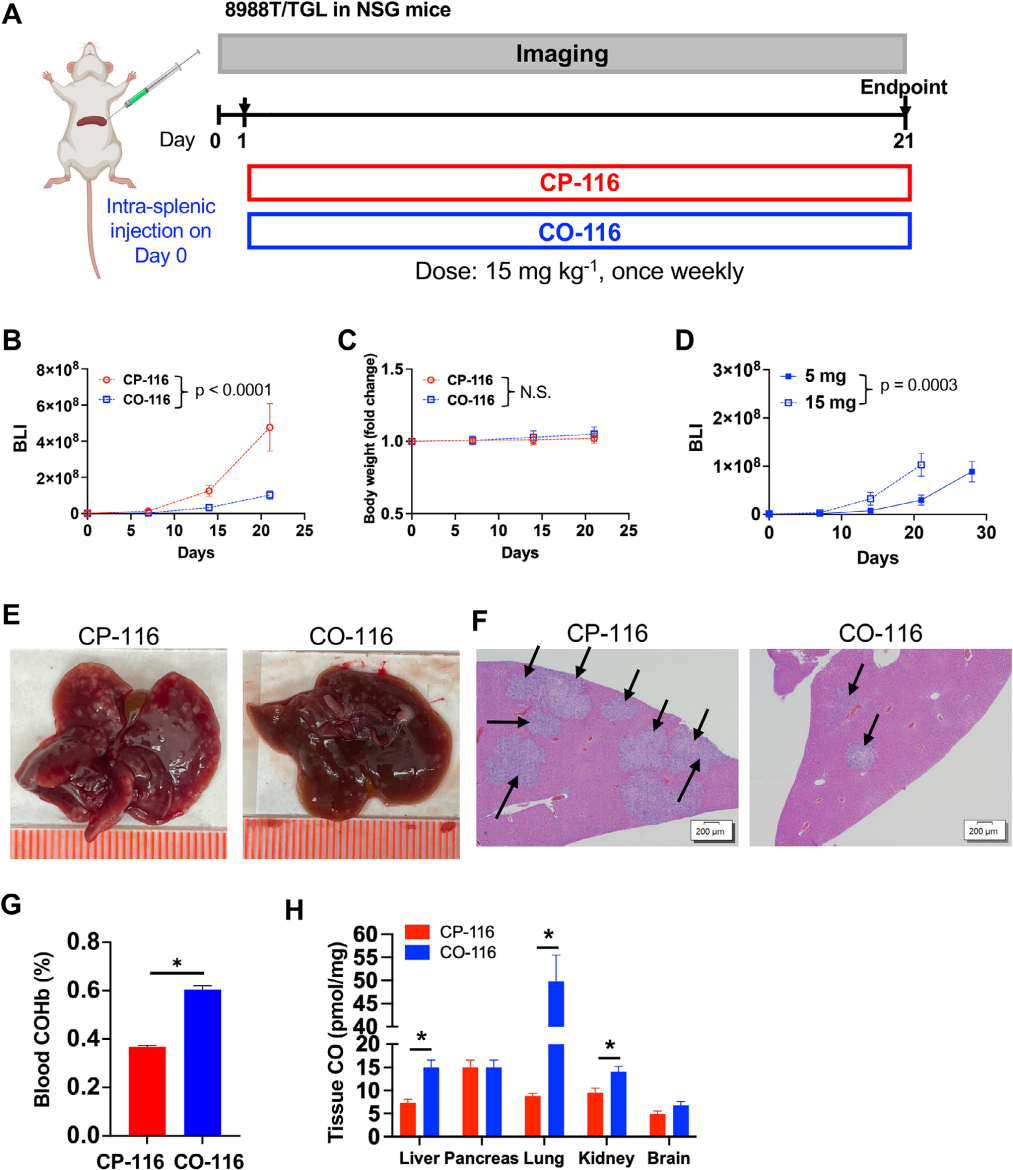
Once‐weekly high‐dose CO‐116 inhibits PDAC liver metastasis but is less effective than more frequent low‐dose treatment. (A) Schematic of the experimental liver metastasis model. NSG mice were intrasplenically injected with 0.05 million 8988T/TGL PDAC cells and randomized into two treatment groups (*n* = 6 per group): **CO‐116**_15 mg (15 mg kg^−1^, IV, once weekly) or **CP‐116**_15 mg control (15 mg kg^−1^, IV, once weekly). (B) Bioluminescent signals of 8988T/TGL cells in mice over 3 weeks. Data are presented as mean ± SEM. Statistical significance was determined using GEE to account for repeated measurements over time. *p* < 0.0001. (C) Longitudinal fold change in body weight. Statistical significance was determined using GEE to account for repeated measurements over time. N.S.: Not significant. (D) Comparison of tumor growth suppression between **CO‐116**_15 mg and **CO‐116**_5 mg. Statistical significance was determined using GEE to account for repeated measurements over time. *p* = 0.0003. (E) Representative gross liver images at necropsy. (F) Representative H&E‐stained liver sections from each group. Arrow: metastasis. Scale bar: 200 µm. (G) COHb levels were measured in terminal blood samples 1 h after the final dose of the drug treatment. Data are presented as mean ± SEM. Statistical significance was determined using a *t*‐test. ^*^ *p* ≤ 0.05. (H) CO levels in tissues harvested 1 h after the final dose of the drug treatment. Data are presented as mean ± SEM. Statistical significance was determined using a *t*‐test. ^*^ *p* ≤ 0.05.

To determine whether schedule—rather than cumulative dose—governs efficacy, we compared regimens delivering the same total weekly dose of CO‐116. NSG mice were randomized one day after tumor inoculation to receive either CO‐116, 15 mg kg^−1^, IV, once weekly, or CP‐116, 15 mg kg^−1^, IV, once weekly (Figure 2A). Longitudinal BLI analyzed on the log scale showed that CO‐116 (15 mg kg^−1^, IV, once weekly) significantly suppressed metastatic growth relative to CP‐116 (Figure 2B; Δslope = −0.054 day^−^^1^, *p* < 0.0001). Treatment was well‐tolerated, with no significant differences in body weight between **CO‐116** and **CP‐116** groups (Figure 2C, *p* = 0.1911). No overt behavioral abnormalities (e.g., changes in activity, grooming, posture, or mobility) were observed in either group during routine daily monitoring. We then benchmarked the once‐weekly regimen against an equally dosed but more frequent schedule—CO‐116, 5 mg kg^−1^, IV, three times per week. Despite identical total weekly dosing (15 mg kg^−1^), the three times per week schedule provided stronger growth control, and the once‐weekly regimen was inferior (Figure 2D; Δslope = +0.053 day^−^^1^ vs 5 mg kg^−1^, thrice‐weekly, *p* = 0.0003), indicating a scheduling advantage for more frequent, lower doses. At necropsy (day 21), macroscopic liver metastases were observed in all CP‐116 (15 mg kg^−1^, once weekly) mice, whereas none were detected in CO‐116 (15 mg kg^−1^, once weekly) mice (Figure 2E). Histopathology corroborated the imaging results, revealing significantly fewer and smaller hepatic metastatic foci in livers from CO‐116 (15 mg kg^−1^, once weekly) versus CP‐116 controls (Figure 2F). These data show that dose scheduling is a key determinant of efficacy: distributing the weekly dose across more frequent, lower administrations yields superior suppression of metastatic outgrowth compared with a single higher weekly dose, even when the total weekly dose is identical.

To examine systemic CO exposure and tissue distribution at the end of treatment, blood and tissues were collected 1 h after the final dose of **CO‐116** or **CP‐116**. **CO‐116** increased blood COHb relative to **CP‐116** while remaining within physiological ranges (Figure 2G). Gas Chromatography (GC) analysis detected CO in multiple organs, with significantly higher tissue CO levels in liver, lung, and kidney in **CO‐116**–treated mice compared with **CP‐116** controls, whereas pancreas and brain showed no significant differences (Figure 2H). These data support controlled systemic CO exposure and organ‐level CO delivery following **CO‐116** administration.

### 2.3 CO‐116 Reduces Lung Metastasis in a TNBC Model and Remains Within Physiological Ranges of CO Exposure

**FIGURE 3:**
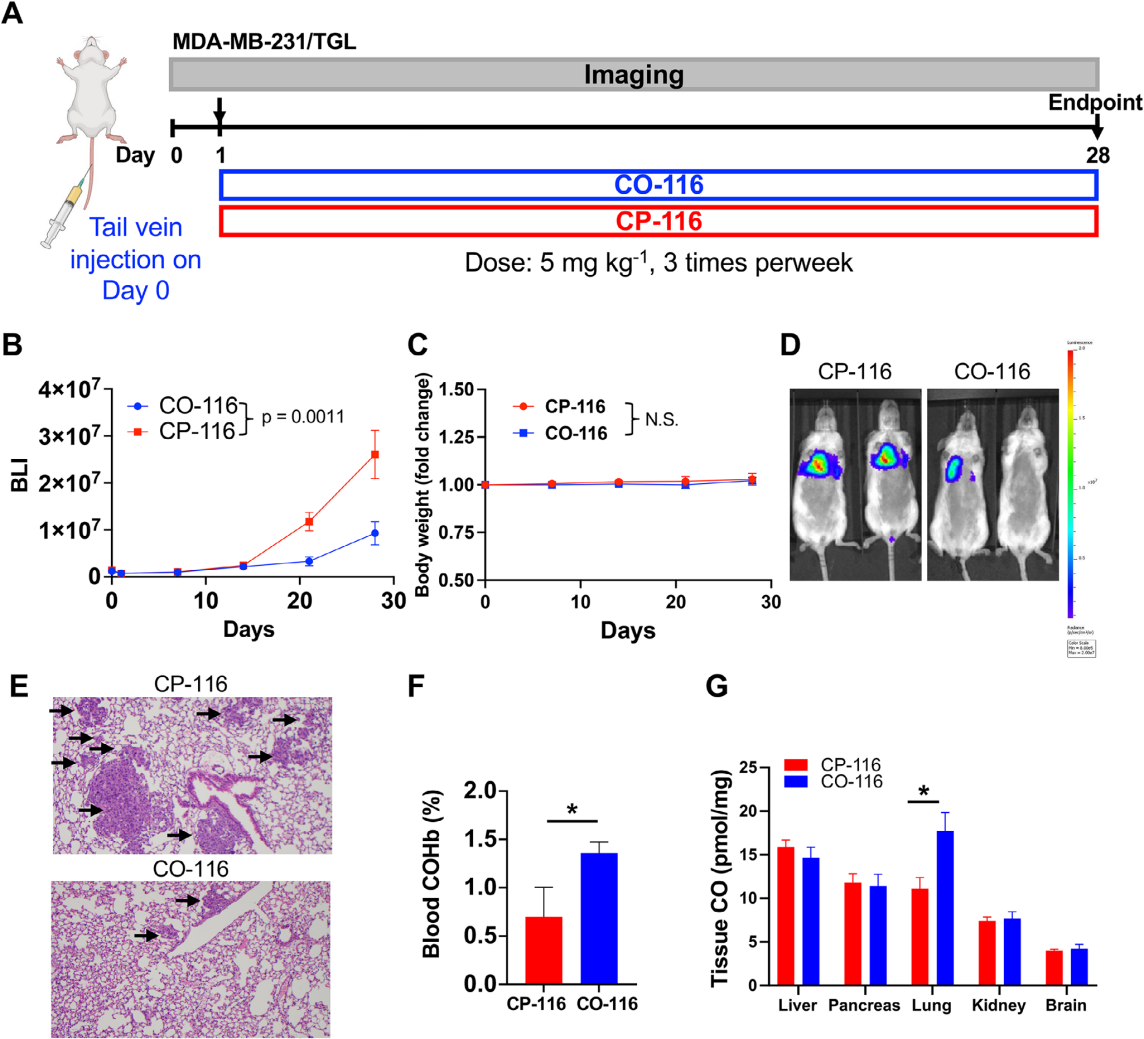
CO‐116 reduces lung metastases in a TNBC tail vein injection model. (A) Schematic of the tail vein lung metastasis model. NSG mice (*n* = 6 per group) were injected with 5 × 10^4^ MDA‐MB‐231/TGL cells and treated with **CO‐116** or **CP‐116** (5 mg kg^−1^, IV, three times per week). (B) Bioluminescent signals of MDA‐MB‐231/TGL cells in mice over time. The final imaging was performed ∼2 h prior to the last drug administration. Statistical significance was determined using GEE to account for repeated measurements over time. Data are presented as mean ± SEM. *p* = 0.0011. (C) Longitudinal fold change in body weight. Statistical significance was determined using GEE to account for repeated measurements over time. Data are presented as mean ± SEM. N.S.: Not significant. (D) Representative BLI images of mice at the end point. (E) Representative H&E‐stained lung sections from each group. Arrow: metastasis. (F) COHb levels were measured from terminal blood samples 1 h after the final dose of the drug treatment. Data are presented as mean ± SEM. Statistical significance was determined using a *t*‐test. ^*^ *p* ≤ 0.05. (G) CO levels in tissues harvested 1 h after the final dose of the drug treatment. Data are presented as mean ± SEM. Statistical significance was determined using a *t*‐test. ^*^ *p* ≤ 0.05.

To evaluate the anti‐metastatic activity beyond PDAC, we tested CO‐116 in an experimental lung metastasis model using MDA‐MB‐231/TGL cells. Tumor cells were delivered by tail vein injection into NSG mice, and mice were randomized 24 h later to CO‐116 (5 mg kg^−1^, IV, three times per week) or CP‐116 on the same schedule (Figure 3A). BLI was performed weekly and analyzed on the log scale with a slope model to quantify metastatic growth kinetics. Longitudinal BLI revealed significantly slower metastatic outgrowth with CO‐116 compared with CP‐116 (Figure 3B; Δslope = −0.048 day^−^^1^, *p* = 0.0011), indicating sustained metastasis control under the three‐times‐a‐week regimen. No significant differences in body weight between **CO‐116** and **CP‐116** groups (Figure 3C; *p* = 0.8203), and no overt behavioral abnormalities were observed in either group during routine daily monitoring. At the endpoint, BLI showed lower photon flux in the lungs from CO‐116‐treated mice relative to CP‐116 controls (Figure 3D). Histopathology corroborated the imaging results, showing fewer and smaller metastatic foci throughout the lung parenchyma after CO‐116 (Figure 3E). No treatment‐related morbidity was observed.

To contextualize exposure, COHb was measured. Values in the CO‐116 group remained within physiological ranges (<3%, typically ∼0.5%–1.5%) and were modestly elevated over CP‐116 controls (Figure 3F). GC analysis of snap‐frozen tissues confirmed increased CO in diseased lungs after CO‐116 (Figure 3G), consistent with systemic delivery and target‐organ exposure under this non‐inhaled dosing paradigm. Together, these data demonstrate that CO‐116 suppresses experimental lung metastasis in TNBC while maintaining physiological CO exposure and acceptable tolerability, extending the anti‐metastatic profile of this metal‐free CO prodrug beyond PDAC.

### 2.4 CO‐116 Downregulates HRG1 and the CYP1B1–SP1 Axis

**FIGURE 4:**
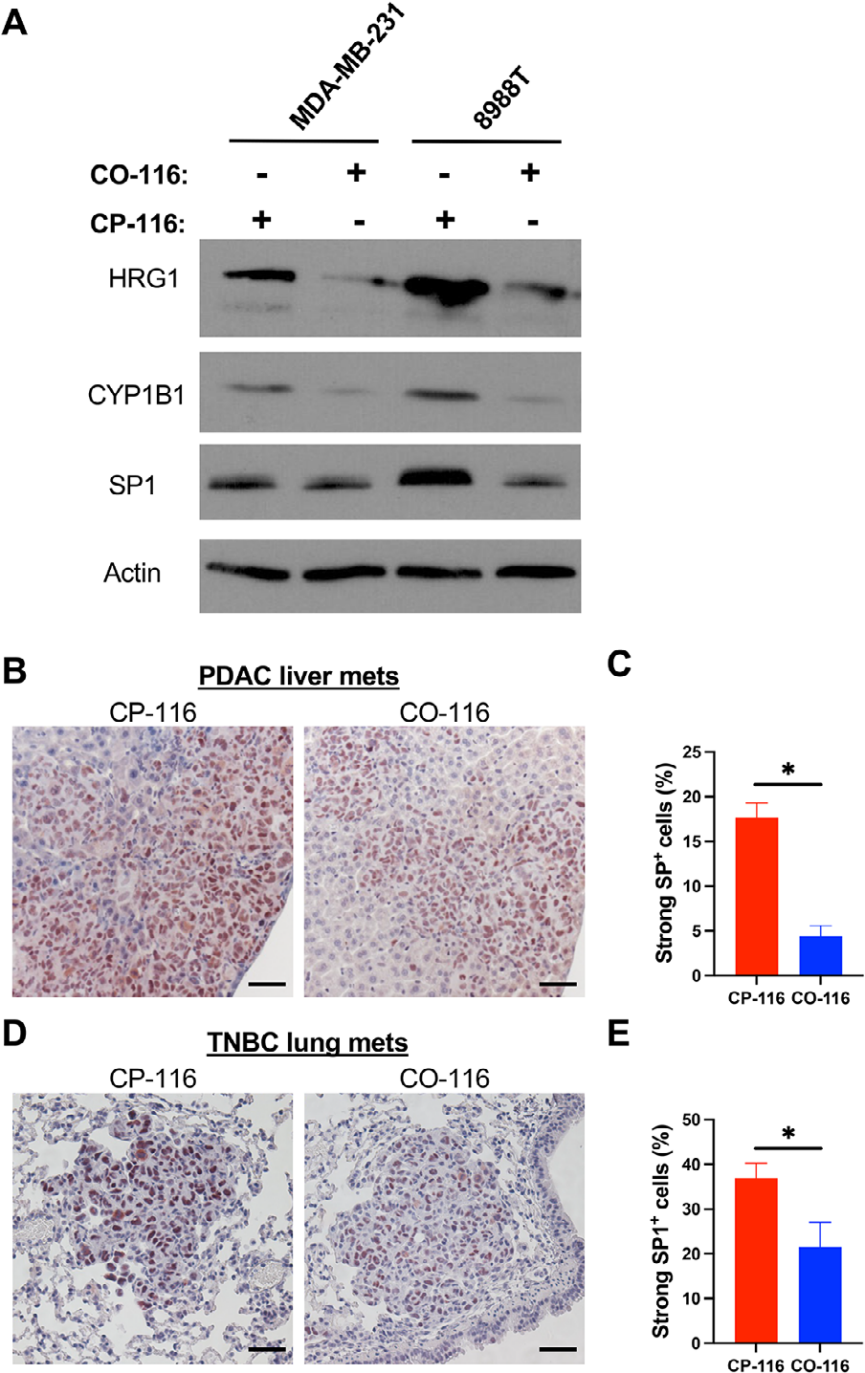
CO‐116 downregulates HRG1, CYP1B1, and SP1. (A) Immunoblot analysis for the protein levels of HRG1, CYP1B1, and SP1 in the indicated cell lines treated with 50 µm CO‐116 or CP‐116 for 5 days. Actin was used as a loading control. (B) Immunohistochemical staining of SP1 in PDAC liver metastases from Figure 1. Data are presented as mean ± SEM. Statistical significance was determined using a *t*‐test. (C) Quantification of strong SP1‐positive tumor cells in the liver metastases. Data are presented as mean ± SEM. Statistical significance was determined using a *t*‐test. ^*^ *p* ≤ 0.05. Scale bar: 50 µm. (D) Immunohistochemical staining of SP1 in TNBC lung metastases from Figure 3. (E) Quantification of strong SP1‐positive tumor cells in the lung metastases. Data are presented as mean ± SEM. Statistical significance was determined using *t*‐test. ^*^ *p* ≤ 0.05. Scale bar: 50 µm.

Given prior evidence that HRG1 transcription is CO‐responsive and linked to metastatic behavior [48], we asked whether **CO‐116** modulates HRG1 and downstream effectors at the protein level. 8988T (PDAC) and MDA‐MB‐231 (TNBC) cells were treated with **CO‐116** or **CP‐116** (50 µm, refreshed daily for 5 days), and cell lysates were analyzed by immunoblot. Across independent experiments, **CO‐116** decreased HRG1 protein, with concordant reductions in the downstream molecules, CYP1B1 and SP1 (Figure 4A).

We next examined these markers in metastatic tumors. In PDAC liver lesions and TNBC lung lesions from mice treated with CO‐116 or CP‐116, IHC revealed predominantly nuclear SP1 staining and fewer SP1‐positive nuclei in the CO‐116 cohorts (Figure 4B,D), with quantitative reductions across multiple regions of interest per specimen (statistics in Figure 4C,E). Where material permitted, SP1 immunoreactivity trended lower in CO‐116–treated lesions, consistent with the in vitro immunoblot data; SP1 provided the most robust in situ readout across cohorts. Together, these findings identify downregulation of HRG1 and attenuation of the CYP1B1–SP1 axis as molecular hallmarks of CO‐116 exposure in vitro and in vivo. These observations motivated the following genetic tests of HRG1 function.

### 2.5 HRG1 Overexpression Promotes Metastatic Traits and Modulates the Response to Low‐Dose CO

**FIGURE 5:**
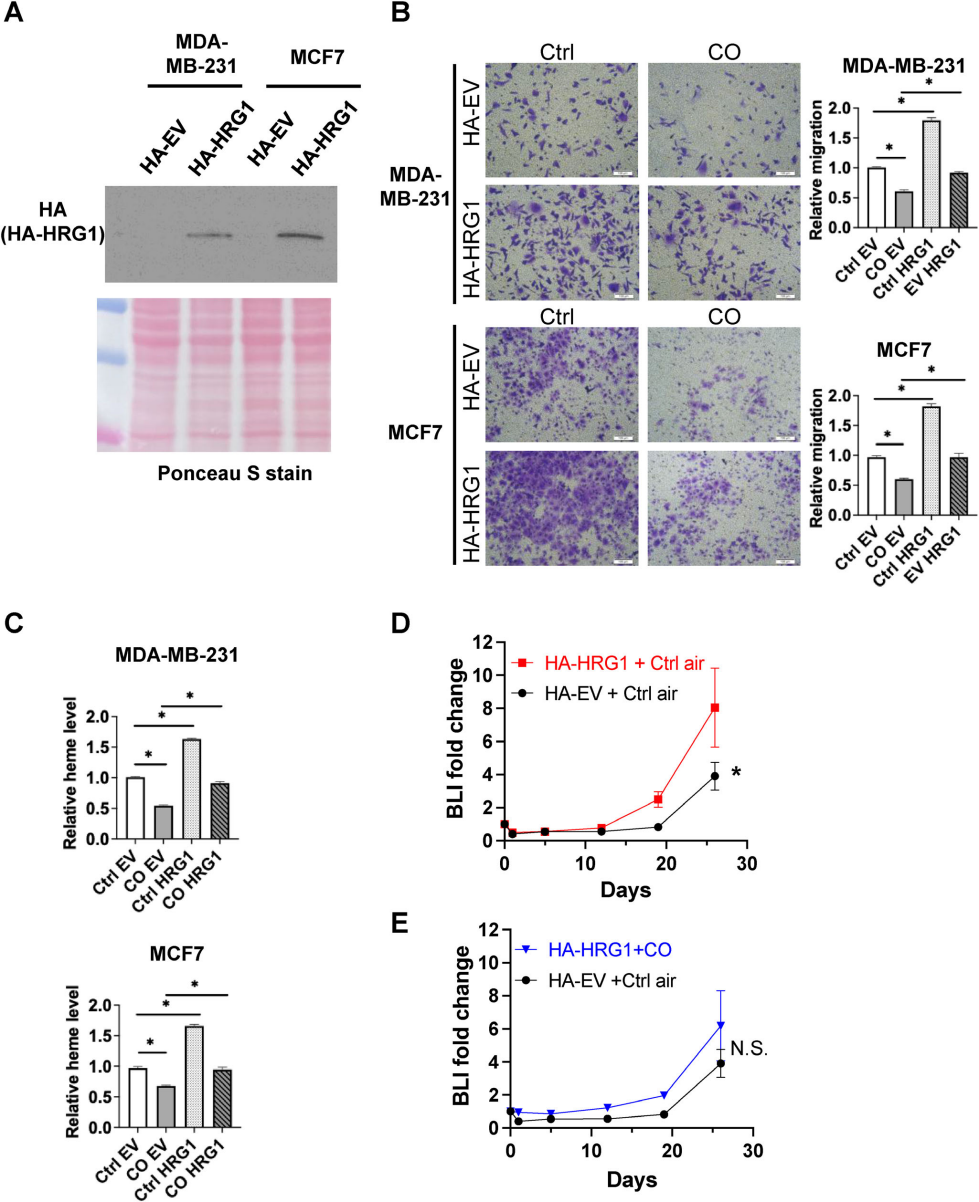
HRG1 drives heme accumulation and counteracts CO‐mediated suppression of migration. (A) Immunoblot analysis for the protein levels of HA‐HRG1 in the indicated cell lines. Ponceau S stain was used as a loading control. EV: empty vector. (B) Migration in MDA‐MB‐231 and MCF7 cells with CO treatment and/or HRG1 overexpression. Statistical significance was determined using two‐way ANOVA with the CO × HRG1 interaction term. MDA‐MB‐231 cells: effects of CO (*p* ≤ 0.001), HRG1 (*p* ≤ 0.001), and their interaction (*p* ≤ 0.001). MCF7 cells: effects of CO (*p* ≤ 0.001), HRG1 (*p* ≤ 0.001), and their interaction (*p* ≤ 0.001). Data represent mean ± SEM (*n* = 3 biological replicates per group). ^*^ *p* ≤ 0.001. (C) Relative intracellular heme levels in MDA‐MB‐231 and MCF7 cells. Statistical significance was determined using two‐way ANOVA with the CO × HRG1 interaction term. MDA‐MB‐231: effects of CO (*p* ≤ 0.001), HRG1 (*p* ≤ 0.001), and their interaction (*p* ≤ 0.001). MCF7 cells: effects of CO (*p* ≤ 0.001), HRG1 (*p* ≤ 0.001), and their interaction (*p* ≤ 0.001). Data represent mean ± SEM (*n* = 3 biological replicates per group). ^*^ *p* ≤ 0.001. (D) Bioluminescent signals of NSG female mice (*n* = 6 per group) receiving MDA‐MB‐231/TGL cells overexpressing HA‐EV or HA‐HRG1 in mice over time. Statistical significance was determined using GEE to account for repeated measurements over time. Data are presented as mean ± SEM. ^*^ *p* ≤ 0.05. (E) Bioluminescent signals of NSG female mice (*n* = 6 per group) receiving MDA‐MB‐231/TGL cells overexpressing HA‐EV or HA‐HRG1 treated with control air or CO (250 ppm, 3 h day^−1^, 5 days a week) over time. Statistical significance was determined using GEE to account for repeated measurements over time. Data are presented as mean ± SEM. N.S.: Not significant.

To test whether elevating HRG1 is sufficient to enhance metastatic behaviors and blunt the response to CO‐based interventions, we generated stable lines expressing HA‐tagged HRG1 (HA‐HRG1) or empty vector (HA‐EV) in MDA‐MB‐231 and MCF7 cells. Immunoblotting confirmed robust overexpression of HA‐HRG1 (Figure 5A). Consistently with our prior finding [48], low‐dose CO significantly reduced migration and intracellular heme in both MDA‐MB‐231 and MCF7 cells overexpressing HA‐EV (Figure 5B,C). In both cell lines, HRG1 overexpression significantly increased baseline migration and intracellular heme levels relative to HA‐EV controls (Figure 5B,C). Two‐way ANOVA demonstrated significant main effects of HRG1 overexpression and CO treatment on both readouts in each cell line (Figure 5B,C, all *p* ≤ 0.001). Importantly, a significant HRG1 × CO interaction was observed, indicating that the effect of CO differs depending on HRG1 status (i.e., CO responsiveness is associated with HRG1 overexpression). Consistent with a heme‐linked motility program, migration strongly correlated with intracellular heme levels across conditions (Figure S2A,B), supporting heme availability as a proximal determinant of motility.

To determine the in vivo consequence of HRG1 gain‐of‐function, we performed an experimental metastasis assay in NSG mice. Mice injected with HA‐HRG1‐overexpressing MDA‐MB‐231/TGL cells exhibited faster increases in bioluminescent signal than HA‐EV controls (Figure 5D). Moreover, fold‐change analysis of longitudinal bioluminescence signals showed no significant difference between the CO–treated HA‐HRG1‐overexpressing group and HA‐EV controls maintained in room air (Figure 5E), indicating that HRG1 overexpression attenuates CO‐mediated metastatic suppression in vivo. Together, these data support that CO suppresses a heme‐dependent migratory program associated with reduced intracellular heme availability and engagement of the HRG1–CYP1B1–SP1 axis.

### 2.6 HRG1 Knockdown Impairs Metastatic Progression in TNBC and PDAC

To test whether loss of HRG1 phenocopies the anti‐metastatic activity observed with CO and **CO‐116**, we generated doxycycline (Dox)–inducible shRNA lines targeting HRG1 (shHRG1#a and shHRG1#b) alongside a control shRNA (shRenilla). Cells were pretreated with Dox for 96 h prior to inoculation. Mice were started on a Dox diet one day prior to tumor cell injection and were maintained on a Dox diet thereafter. Metastatic growth was quantified by longitudinal BLI and analyzed on the log scale with a slope model.

**FIGURE 6:**
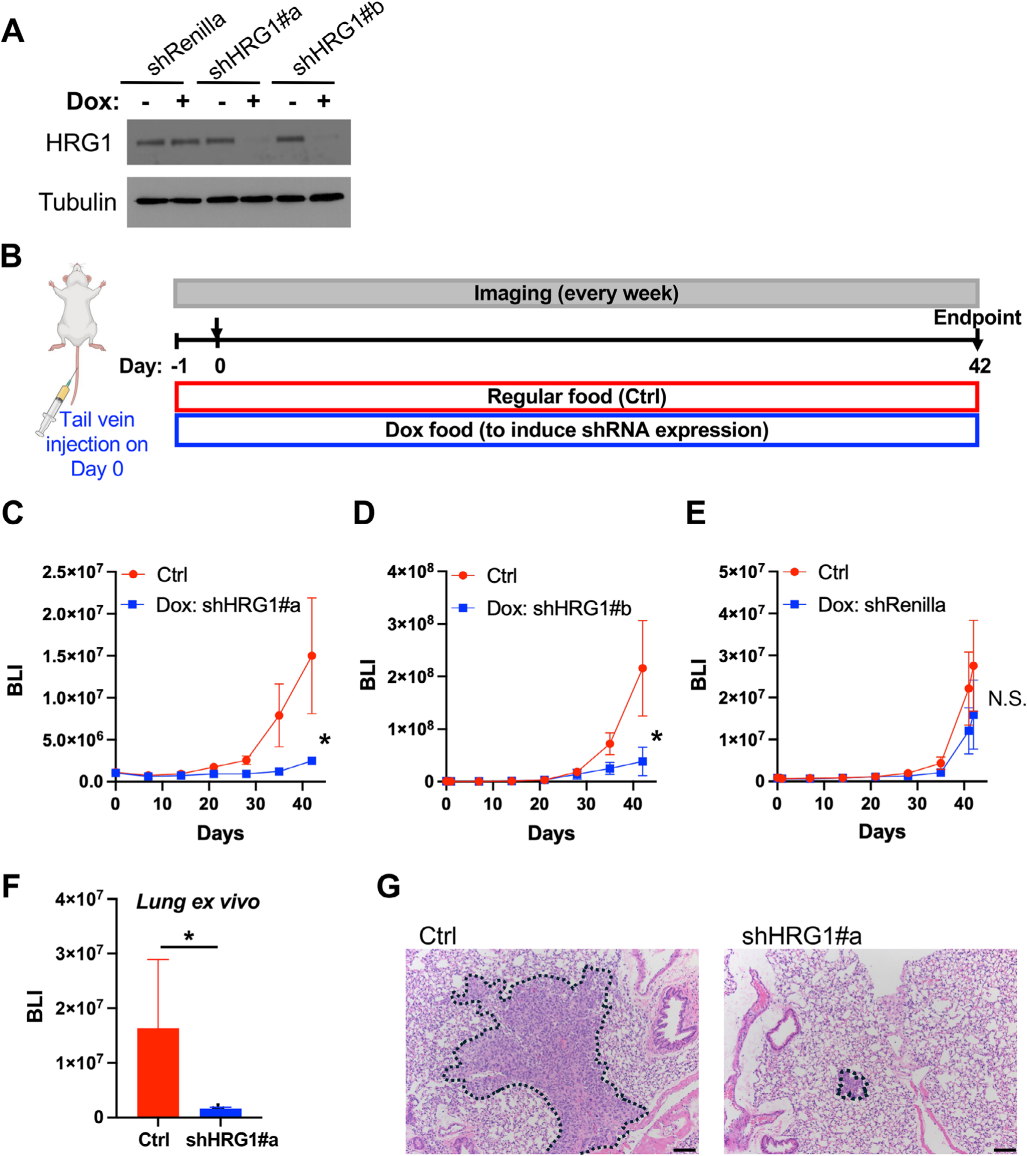
shRNA knockdown of HRG1 suppresses TNBC lung metastasis in vivo. (A) Immunoblot analysis for the protein levels of HRG1 in the Dox‐pretreated MDA‐MB‐231/TGL/tet‐O‐shRenilla (#713), MDA‐MB‐231/TGL/tet‐O‐shHRG1#a, and MDA‐MB‐231/TGL/tet‐O‐shHRG1#b cells. Tubulin was used as a loading control. (B) Schematic of the tail vein lung metastasis model. Dox‐pretreated MDA‐MB‐231/TGL/tet‐O‐shRenilla (#713), MDA‐MB‐231/TGL/tet‐O‐shHRG1#a, and MDA‐MB‐231/TGL/tet‐O‐shHRG1#b cells were injected into NSG mice that started a doxycycline diet one day before tumor cell injection. The untreated control cells were injected into NSG mice that were on a regular diet. (C, D, E) Bioluminescent signals of NSG female mice (*n* = 3 per group for shHRG1#a, *n* = 4 per group for shHRG1#b and shRenilla) over the course of the 42 days. Statistical significance was determined using GEE to account for repeated measurements over time. Data are presented as mean ± SEM. ^*^ *p* ≤ 0.05. N.S.: Not significant. shHRG1#a: *p* < 0.0001 in (C); shHRG1#b: *p* = 0.0066 in (D); shRenilla, *p* = 0.0726 in (E). (F) Ex vivo BLI of lungs at the endpoint. Data are presented as mean ± SEM. Statistical significance was determined using a *t*‐test. ^*^ *p* ≤ 0.05. (G) Representative H&E‐stained lung tissues. Circle: metastasis. Scale bar: 200 µm.

In the TNBC model (MDA‐MB‐231/TGL, tail vein metastasis assay), Dox‐induced HRG1 knockdown markedly slowed metastatic outgrowth relative to matched no‐Dox controls. For shHRG1#a, the BLI growth rate was significantly reduced (*p* < 0.0001), and for shHRG1#b, the reduction was likewise significant (*p* = 0.0066) (Figure 6A–D). In contrast, Dox had no effect in shRenilla controls (*p* = 0.0726) (Figure 6E). Endpoint analyses corroborated the kinetic results: ex vivo BLI and histology showed fewer and smaller lung lesions in the Dox–treated shHRG1 groups compared with their no‐Dox counterparts (Figure 6F,G, and data not shown).

**FIGURE 7:**
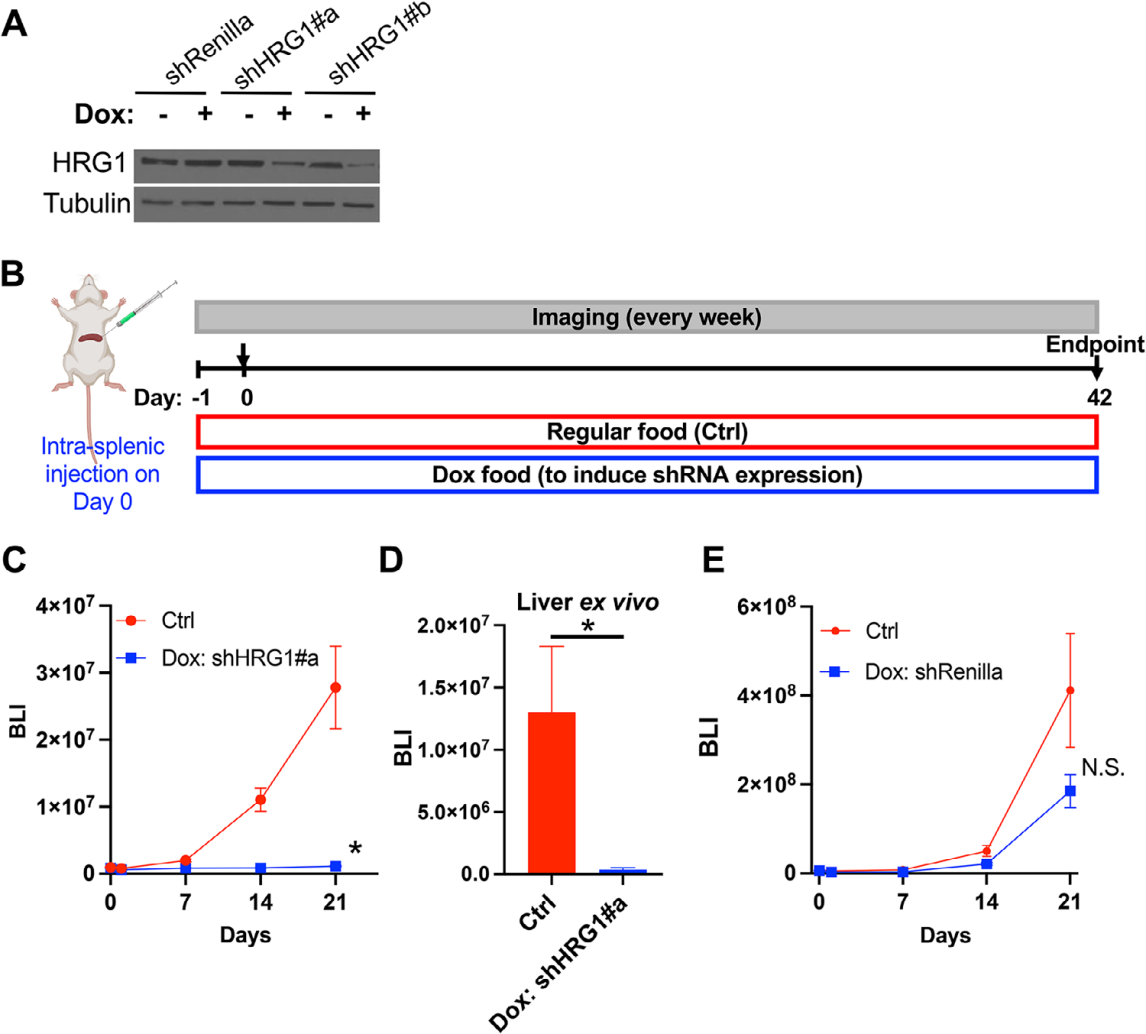
shRNA knockdown of HRG1 suppresses PDAC liver metastasis in vivo. (A) Immunoblot analysis for the protein levels of HRG1 Dox‐pretreated 8988T/TGL/tet‐O‐shRenilla (#713), 8988T/TGL/tet‐O‐shHRG1#a, and 8988T/TGL/tet‐O‐shHRG1#b cells. Tubulin was used as a loading control. (B) Schematic of the experimental design. Dox‐pretreated 8988T/TGL/tet‐O‐shHRG1#a, and 8988T/TGL/tet‐O‐shRenilla (#713) cells were injected into NSG mice that started a doxycycline diet one day before tumor injection (*n* = 7 per group). The untreated control 8988T/TGL/tet‐O‐shRenilla (#713) cells were injected into NSG mice that were on a regular diet (*n* = 7 per group). (C) Bioluminescent signals from mice receiving 8988T/TGL/tet‐O‐shHRG1#a over the course of the 21 days. Statistical significance was determined using GEE to account for repeated measurements over time. Data are presented as mean ± SEM. ^*^ *p* ≤ 0.05. (D) Ex vivo BLI of livers at the endpoint. (E) Bioluminescent signals from mice receiving 8988T/TGL/tet‐O‐shRenilla (#713) over the course of the 21 days. Statistical significance was determined using GEE to account for repeated measurements over time. Data are presented as mean ± SEM. N.S.: Not significant.

We next assessed HRG1 loss in a PDAC liver metastasis model (8988T/TGL, intrasplenic injection). Knockdown of HRG1 in 8988T/TGL by Dox‐inducible shHRG1#a produced a pronounced deceleration of metastatic growth, with an estimated change in log‐scale slope of Δslope = −0.145 day^−^^1^ versus no‐Dox control (*p* < 0.0001) (Figure 7A–C). Consistently, ex vivo liver BLI at endpoint demonstrated significantly lower tumor burden in the Dox–treated shHRG1a group compared with no‐Dox controls (Figure 7D; *p* < 0.0001). As in the TNBC model, Dox treatment of shRenilla controls had no measurable effect on metastatic growth (Figure 7E; Δslope = −0.009 day^−^^1^, *p* = 0.3641). Across models, HRG1 knockdown reduced metastatic expansion without overt treatment‐related morbidity. Together with the ant‐metastatic efficacy of CO prodrugs (Sections 2.1–2.3) and the protein‐level changes of HRG1 (Section 2.4), these convergent gain‐ and loss‐of‐function results establish HRG1 as a functional mediator within the CO‐responsive program that governs metastatic progression.

### 2.7 CO‐116 Suppresses Metastatic Progression in an Immunocompetent TNBC Mouse Model

**FIGURE 8:**
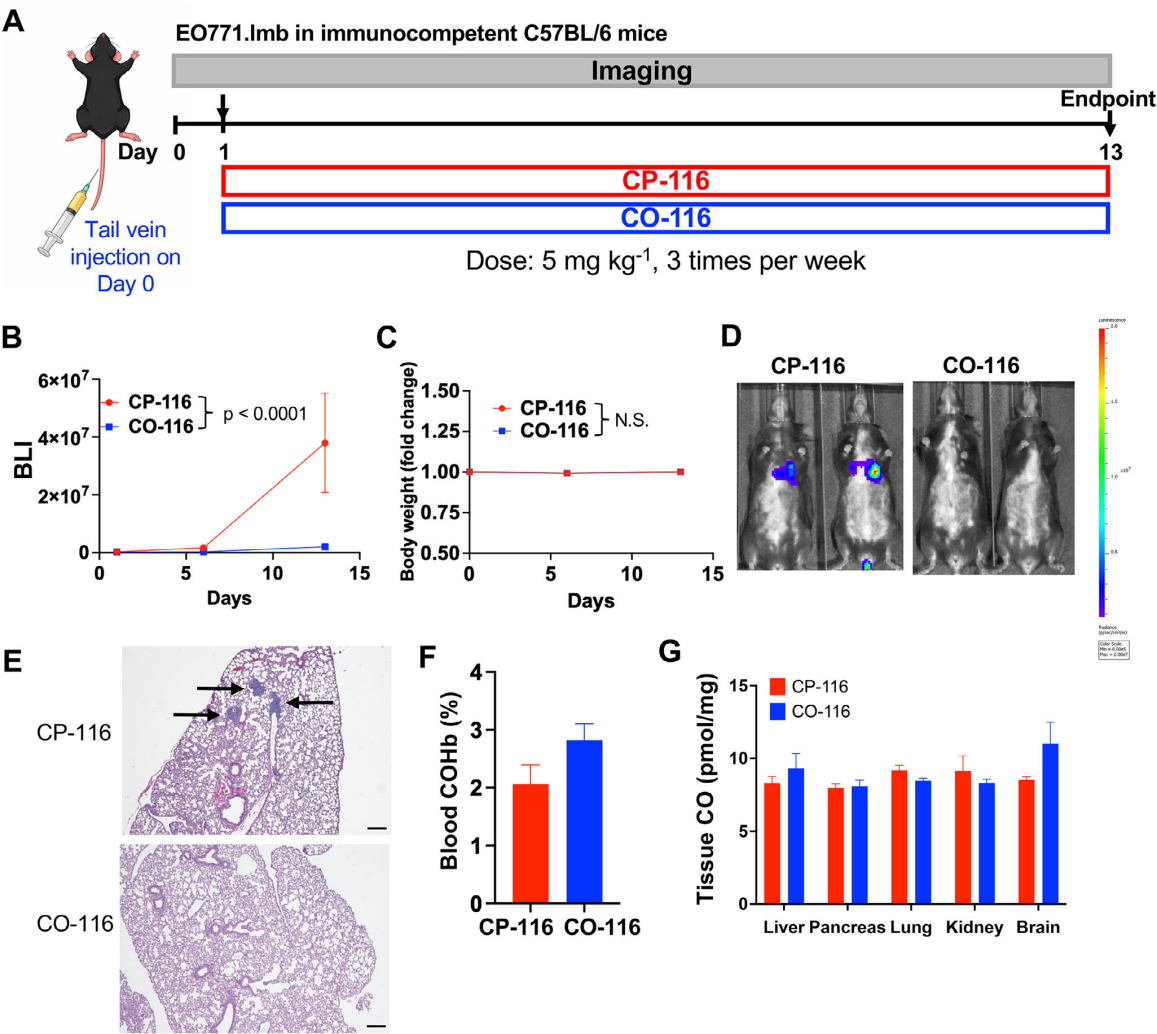
CO‐116 efficacy and CO exposure in immunocompetent mice. (A) Experimental scheme for the EO771.lmb syngeneic TNBC metastasis model in C57BL/6 female mice treated with CP‐116 or CO‐116 (5 mg kg^−1^, IV, three times per week; *n* = 5 per group). (B) Bioluminescent signals of EO771.lmb cells in mice over 2 weeks. The final imaging was performed ∼2 h prior to the last drug administration. Statistical significance was determined using GEE to account for repeated measurements over time. Data are presented as mean ± SEM. *p* ≤ 0.0001. (C) Longitudinal fold change in body weight. Statistical significance was determined using GEE to account for repeated measurements over time. Data are presented as mean ± SEM. N.S.: Not significant. (D) Representative BLI images of mice at the end point. (E) Representative H&E‐stained lung sections from each group. Arrow: metastasis. Scale bar: 200 µm. (F) COHb levels were measured from terminal blood samples 1 h after the final dose of the drug treatment. Data are presented as mean ± SEM. Statistical significance was determined using a *t*‐test. *p* = 0.1227. (G) CO levels in tissues harvested 1 h after the final dose of the drug treatment. Data are presented as mean ± SEM. Statistical significance was determined using a *t*‐test.

To determine whether the anti‐metastatic efficacy of **CO‐116** is preserved in the presence of an intact immune system, we evaluated **CO‐116** in a syngeneic TNBC metastasis model using EO771.lmb cells in C57BL/6 female mice. Mice were treated with CP‐116 or CO‐116 (5 mg kg^−1^, IV, three times per week) (Figure 8A), and metastatic burden was monitored longitudinally by BLI, with the final imaging performed ∼2 h prior to the last drug administration. **CO‐116** significantly slowed metastatic progression compared with the **CP‐116** control (Figure 8B). Treatment was well‐tolerated, with no significant differences in body weight over time between groups (Figure 8C, *p* = 0.6447). No overt behavioral abnormalities were observed in either group during routine daily monitoring. At endpoint, BLI showed lower photon flux in the lungs from the **CO‐** **116** group relative to the **CP‐** **116** group (Figure 8D) and histopathology suppted the BLI results (Figure 8E). Blood COHb and tissue CO levels measured 1 h after the final dose remained within physiological ranges and did not differ significantly between **CO‐116**– and **CP‐116**–treated mice, indicating controlled systemic CO exposure in immune‐intact hosts (Figure 8F,G; Table 1). Collectively, these data demonstrate that **CO‐116** retains anti‐metastatic efficacy in an immunocompetent setting without evidence of overt systemic toxicity.

**TABLE 1:** Tissue carbon monoxide (CO) levels in an immunocompetent triple‐negative breast cancer (TNBC) model following CP‐116 versus CO‐116 treatment.

Tissue CO (pmol mg^−1^)	All data (*n* = 10)	Treatment (5 mg kg^−1^, 3 times week^−1^ for 2 weeks)		—
		CP‐116 (*n* = 5)	CO‐116 (*n* = 5)	*p*‐value ^a^
Liver	—	—	—	0.378
Mean (SD)	8.8 (1.7)	8.3 (1.1)	9.3 (2.2)	—
Median (Q1, Q3)	8.7 (7.4, 10)	8.2 (7.4, 9)	9.2 (7.4, 10)	—
Min, Max	7.1, 12.5	7.1, 9.6	7.2, 12.5	—
Pancreas	—	—	—	0.853
Mean (SD)	8.0 (0.8)	8.0 (0.6)	8.1 (1.0)	—
Median (Q1, Q3)	8.1 (7.4, 8)	8.3 (7.4, 8)	7.8 (7.4, 8)	—
Min, Max	7.1, 9.6	7.2, 8.6	7.1, 9.6	—
Lung	—	—	—	0.120
Mean (SD)	8.8 (0.7)	9.2 (0.8)	8.5 (0.4)	—
Median (Q1, Q3)	8.8 (8.3, 9)	9.0 (8.9, 9)	8.4 (8.3, 9)	—
Min, Max	8.0, 10.5	8.2, 10.5	8.0, 9.0	—
Kidney	—	—	—	0.454
Mean (SD)	8.7 (1.7)	9.1 (2.3)	8.3 (0.6)	—
Median (Q1, Q3)	8.3 (7.7, 9)	8.4 (7.7, 9)	8.2 (8.1, 8)	—
Min, Max	7.2, 13.1	7.2, 13.1	7.4, 9.2	—
Brain	—	—	—	0.136
Mean (SD)	9.8 (2.6)	8.5 (0.5)	11.0 (3.3)	—
Median (Q1, Q3)	8.6 (8.2, 10)	8.3 (8.2, 9)	9.8 (8.6, 13)	—
Min, Max	7.9, 15.9	8.1, 9.3	7.9, 15.9	—

^a^ Two‐Sample *t*‐test.

### 2.8 Dose‐Dependent Systemic CO Exposure Following CO‐116 Administration in Healthy Mice

To determine whether **CO‐116** exhibits dose‐dependent systemic exposure and tissue distribution independent of tumor burden, we performed a pharmacokinetic analysis in non‐tumor‐bearing C57BL/6 mice. Animals received a single intravenous (IV) injection of **CO‐116** at 0 (untreated), 5, or 15 mg kg^−1^, and blood and tissues were collected 1 h after dosing for COHb measurement and GC analysis of tissue CO. COHb levels increased in a dose‐dependent manner while remaining within physiological ranges, and COHb correlated positively with administered dose (Figure S3, r = 0.53, *p* = 0.039), supporting dose‐dependent systemic CO exposure despite inter‐individual variability. GC analysis detected CO across multiple tissues (liver, lung, kidney, pancreas, and brain) in untreated and **CO‐116**‐treated mice, with no significant increase in bulk tissue CO (Table 2). Because tissue CO was readily detectable in untreated mice and did not significantly increase following these **CO‐116** dosing at this time point, the measured bulk tissue CO likely reflects the dominant endogenous CO pool—primarily CO bound to hemoproteins—rather than net accumulation of exogenous CO from the prodrug. Together, these findings indicate controlled systemic CO exposure at therapeutically effective dosing without overt tissue‐specific accumulation. Of note, these analyses are intended to define distributional trends, with broader considerations for interpreting COHb and tissue CO metrics in relation to pharmacological outcomes discussed below.

**TABLE 2:** Tissue CO levels in healthy immunocompetent mice following single‐dose CO‐116 administration.

Tissue CO (pmol mg^−1^)	All data (*n* = 15)	CO‐116 dose (mg kg^−1^)			*p*‐value ^a^
		0 (*n* = 5)	5 (*n* = 5)	15 (*n* = 5)	
Liver	—	—	—	—	0.231
Mean (SD)	8.8 (1.2)	8.3 (0.7)	8.3 (0.8)	9.8 (1.6)	—
Median (Q1, Q3)	8.5 (7.7, 9)	8.2 (8.0, 9)	8.1 (7.7, 9)	9.9 (9.1, 10)	—
Min, Max	7.5, 12.0	7.5, 9.3	7.5, 9.1	7.7, 12.0	—
Pancreas	—	—	—	—	0.530
Mean (SD)	8.4 (1.4)	8.1 (1.3)	9.1 (1.8)	7.9 (0.8)	—
Median (Q1, Q3)	7.6 (7.2, 9)	7.3 (7.1, 9)	8.7 (7.4, 11)	7.6 (7.5, 8)	—
Min, Max	7.1, 11.0	7.1, 9.5	7.2, 11.0	7.1, 9.1	—
Lung	—	—	—	—	0.327
Mean (SD)	9.4 (1.9)	8.7 (0.9)	8.7 (1.6)	10.6 (2.4)	—
Median (Q1, Q3)	9.2 (8.0, 11)	8.2 (8.1, 9)	8.1 (7.6, 10)	10.7 (10.1, 11)	—
Min, Max	7.1, 14.2	8.0, 10.1	7.1, 10.8	7.5, 14.2	—
Kidney	—	—	—	—	0.916
Mean (SD)	8.9 (1.2)	8.9 (0.5)	8.6 (2.0)	9.1 (0.8)	—
Median (Q1, Q3)	8.7 (7.9, 9)	9.1 (8.5, 9)	7.7 (7.6, 8)	9.5 (8.7, 10)	—
Min, Max	7.5, 12.1	8.3, 9.4	7.5, 12.1	7.9, 9.6	—
Brain	—	—	—	—	0.488
Mean (SD)	9.2 (2.2)	8.3 (1.7)	9.5 (2.5)	9.8 (2.4)	—
Median (Q1, Q3)	8.4 (7.6, 11)	7.6 (7.6, 8)	8.4 (7.6, 12)	9.3 (9.1, 9)	—
Min, Max	7.2, 13.9	7.2, 11.3	7.3, 12.5	7.4, 13.9	—

^a^ One‐way analysis of means (not assuming equal variances)

## 3 Discussion

Metastatic recurrence remains the leading cause of mortality in PDAC and TNBC, yet few therapies are designed specifically to suppress metastatic outgrowth while maintaining minimal systemic toxicity. Here, we demonstrate that the metal‐free CO prodrug, **CO‐116**, suppresses metastatic progression in vivo across experimental PDAC and TNBC models in both immunodeficient and immunocompetent settings, while maintaining physiological COHb levels and favorable tolerability.

Previous studies, including our own, showed that low‐dose inhaled CO can inhibit primary tumor growth and metastatic outgrowth, implicating modulation of heme‐dependent signaling pathways as a potential mechanism [46, 47, 48]. However, these studies relied on inhaled CO or metal‐based CORMs and did not address whether a metal‐free CO prodrug could reproduce these effects in vivo, nor whether the proposed HRG1–heme axis functionally mediates metastatic suppression in vivo. This knowledge gap is particularly relevant for translation, as inhaled CO poses logistical and safety challenges for chronic or adjuvant use, and metal‐based CORMs carry risks of metal‐associated toxicity and CO‐independent effects [22, 23, 24, 25, 26, 27, 28, 29, 30, 31, 32, 33, 34]. Our data demonstrate that CO‐116 recapitulates anti‐metastatic efficacy while overcoming the translational limitations of inhaled gas delivery and metal‐based CO donors.

Importantly, head‐to‐head schedule comparisons revealed that exposure pattern, not cumulative dose alone, is a key determinant of efficacy: once‐weekly **CO‐116** at 15 mg kg^−1^ reduced tumor growth relative to control but was inferior to the same total weekly dose divided into three administrations. This identifies the dosing schedule as an actionable lever to optimize benefit without increasing total weekly dose.

Evaluation of a structurally distinct scaffold, **CO‐103**, further supports a class effect of metal‐free CO prodrugs. **CO‐103** and **CO‐116** represent two chemically distinct classes of CO prodrugs [49, 50]. Despite these structural differences, they exhibit comparable CO‐release kinetics in biological matrices, with similar half‐lives in plasma (1.29 h for **CO‐103** and 1.48 h for **CO‐116**) [52]. The compounds also display broadly similar plasma protein binding (92% for **CO‐103** versus 84% for **CO‐116**) [52]. **CO‐103** likewise reduced metastatic burden in the PDAC model, supporting a class effect of metal‐free CO prodrugs and suggesting that controlled CO release—rather than scaffold‐specific chemical features—is the primary driver of biological activity. Although intravenous **CO‐103** was associated with local tail‐biting behavior suggestive of injection site irritation (not observed with **CP‐103** or **CO‐116**), this did not diminish anti‐metastatic efficacy. Based on its favorable local tolerability and suitability for repeated dosing, **CO‐116** was selected for detailed mechanistic and schedule‐optimization studies. Inclusion of **CO‐103** strengthens the conclusion that metastasis suppression is not restricted to a single scaffold.

Mechanistically, our data position HRG1 as a functional component within a CO‐responsive program influencing metastatic progression. **CO‐116** reduced HRG1, CYP1B1, and SP1 in cultured cells, and SP1 immunoreactivity was reduced in metastatic lesions in mice treated with **CO‐116**. Gain‐of‐function studies demonstrated that HRG1 overexpression increased intracellular heme levels and enhanced metastatic traits, whereas loss‐of‐function slowed metastatic expansion across TNBC and PDAC models without overt morbidity. Importantly, migration strongly correlated with intracellular heme levels across conditions, supporting heme availability as a proximal determinant of motility. These findings indicate that CO suppresses a heme‐dependent metastatic program that includes HRG1, CYP1B1, and SP1. A limitation of this study is that direct in situ quantification of HRG1 knockdown within metastatic lesions was not feasible. HRG1‐knockdown metastatic foci were extremely small, precluding reliable microdissection for qPCR analysis, and commercially available HRG1 antibodies were not compatible with IHC application under our experimental conditions.

An important next step will be to determine whether metastatic suppression persists after **CO‐116** withdrawal. Because **CO‐116** modulates a heme‐linked signaling program rather than inducing overt cytotoxicity, defining the durability and reversibility of response will be essential for optimizing dosing schedules. Extended post‐treatment monitoring studies focused on recurrence, rather than suppression during active therapy, will be required to address this question. Such experiments were beyond the scope of the present proof‐of‐concept study but represent a critical direction for future investigation.

While a systematic evaluation of organ‐specific drug distribution—in both healthy and tumor‐bearing mice, and in untreated versus CO‐treated hosts—represents a logical step in studying a typical small molecule drug, such studies with CO has been challenging across the CO field. This difficulty likely reflects multiple factors, including the practical limitations of real‐time tissue CO sampling, the volatility and low aqueous solubility of CO, and context‐dependent shifts in hemoglobin–CO binding influenced by pH, metabolites (e.g., diphosphoglycerate), and oxygen partial pressure. In many settings, measured tissue CO levels are strongly influenced by local hemoprotein abundance and distribution [53, 54]. Furthermore, the tetrameric and allosteric nature of hemoglobin binding with CO means that identical COHb percentages may correspond to different underlying exposure dynamics and tissue delivery contexts, as analyzed in detail previously [8, 9, 55, 56]. Partitioning of CO between COHb and a given target under the local conditions becomes a critical factor in determining target engagement. Detailed discussions of various factors affecting CO target engagement, including mathematical modeling, have also been reported [57]. Consistent with these complexities, it is broadly recognized that COHb levels do not reliably predict clinical outcomes, and the route of administration makes a difference in terms of what a given COHb number means, as systematically discussed in recent publications [9, 55, 57, 58]. Overall, correlating COHb and tissue CO measurements with pharmacological efficacy remains technically and conceptually challenging, underscoring the importance of integrating exposure metrics with downstream pharmacodynamic readouts.

Together, this work advances CO‐based anti‐metastatic therapy from an inhaled gas paradigm to a non‐inhaled, metal‐free prodrug strategy while preserving mechanistic continuity through a heme‐centered program involving HRG1, CYP1B1, and SP1. This work decouples therapeutic CO delivery from the logistical constraints of inhaled gas and from potential metal‐associated liabilities reported for some classical donors. It identifies schedule dependence as a modifiable determinant of efficacy and establishes a mechanistic framework linking pharmacologic CO exposure to heme‐dependent metastatic signaling. Future studies should define upstream regulators of HRG1, extend toxicology and pharmacokinetic/pharmacodynamic modeling to optimize clinical translation, and develop clinically deployable assays for HRG1/SP1 levels as biomarkers. Route and formulation optimization to enable outpatient administration will further enhance translational feasibility [9, 55].

## 4 Materials and Methods

### 4.1 Cell Lines and Plasmids

#### 4.1.1 Cell Lines, Authentication, and the Rationale for the Use of the Cell Lines

8988T/TGL (PDAC) was derived from parental PaTu 8988T (DSMZ ACC‐162; RRID: CVCL_1847) and generated in‐house as described [48]. Cell line identity was confirmed by short tandem repeat (STR) authentication. MDA‐MB‐231/TGL, a gift from Dr. Joan Massagué’s laboratory [59], was derived from the parental MDA‐MB‐231 (ATCC HTB‐26; RRID: CVCL_0062). Historical purchase dates for the parental vials cannot be retrieved. All cell lines tested mycoplasma‐negative. Cells were cultured in DMEM (Corning, 10‐013‐CV) supplemented with 10% fetal bovine serum (FBS), additional 2 mm L‐glutamine, and 1% penicillin/streptomycin. PaTu 8988T/TGL was chosen as the PDAC model for its reliable liver colonization after intrasplenic injection and its compatibility with longitudinal BLI, mirroring a common clinical site of PDAC spread. MDA‐MB‐231/TGL and EO771.lmb [60] were selected as the TNBC models for their established propensity to form lung metastases after tail vein injection and its suitability for longitudinal BLI, enabling sensitive slope‐based growth analyses.

#### 4.1.2 Plasmids

pSingle‐tTs‐shRenilla (#713) was constructed as follows. The pSingle‐tTS empty vector (Clontech Laboratories) was digested with *HindIII* (New England Biolabs; Cat. #R0104) and *XhoI* (New England Biolabs; Cat. #R0146) following the manufacturer's instructions. Complementary oligonucleotides OND1630 (5′‐AGCTTACGCGTAAAAAAGTAGATAAGCATTATAATTCCTATCTCTTGAATAGGAATTATAATGCTTATCTACC‐3′) and OND1631 (5′‐TCGAGGTAGATAAGCATTATAATTCCTATTCAAGAGATAGGAATTATAATGCTTATCTACTTTTTTACGCGTA‐3′) were synthesized by MilliporeSigma. The oligonucleotides were mixed at a 1:1 molar ratio in 1X TE buffer (10 mm Tris‐HCl, 1 mm EDTA, pH 8.0) at a final concentration of 100 µm each. Annealing was performed in a PCR thermocycler (Eppendorf) with the following program: 95°C for 30 s, 72°C for 2 min, 37°C for 2 min, and 25°C for 2 min. The resulting double‐stranded DNA fragment, which carried cohesive ends compatible with *HindIII* and *XhoI* restriction sites, was directly ligated into the digested vector using T4 DNA Ligase (New England Biolabs; Cat. #M0202) according to the manufacturer's protocol. The ligation mixture was transformed into competent *E. coli* cells using heat shock. Positive colonies were screened, and plasmid DNA was purified and sequence‐verified (Psomagen) prior to mammalian cell transfection.

pSingle‐tTS‐shHRG1#a, pSingle‐tTS‐shHRG1#b, pcDNA3/HA‐EV (empty control vector), and pcDNA3/HA‐HRG1 were kindly provided by Dr. Rosemary O'Connor [61]. pSingle‐tTS‐shRenilla(#713), pSingle‐tTS‐shHRG1#a, pSingle‐tTS‐shHRG1#b, pcDNA3/HA‐EV, and pcDNA3/HA‐HRG1 were transfected into MCF7/TGL, MDA‐MB‐231/TGL, and 8988T/TGL cells using Lipofectamine 3000 and P3000 Reagent (Invitrogen, Carlsbad, CA, USA) according to the manufacturer's instructions. Transfected cells were selected in 0.8 mg/mL G418 (Thermo Fisher Scientific) for 1 week and maintained in 0.4 mg/mL G418 thereafter.

### 4.2 Preparation of CO Prodrugs for In Vitro and In Vivo Experiments

#### 4.2.1 Compound

**CO‐116**, **CP‐116**, **CO‐103**, and **CP‐103** were synthesized as described previously [49, 50].

#### 4.2.2 In Vitro Stocks and Working Solutions

CO‐116 and CO‐103 stock solutions in DMSO (10–20 mm) should be prepared fresh immediately before use. Only CP‐116 and CP‐103 stock solutions in DMSO may be stored at −20°C (20 mm, up to 1 month). If CP‐103 or CP‐116 dissolves slowly, gently warm the DMSO stock to 50°C–60°C, then allow to return to room temperature before dilution. Do not heat **CO‐103 or CO‐116** during stock preparation, as warming can prematurely trigger CO release. Working solutions were made by diluting stocks into FBS‐containing medium to 50 µm (final DMSO 0.05–0.1% v/v).

#### 4.2.3 In Vivo Dosing Solutions

For injections, **CO‐116**, **CP‐116**, **CO‐103**, and **CP‐103** were freshly dissolved in DMSO at 10 mg/mL and mixed 1:1 (v/v) with Solutol. Immediately before dosing, the DMSO/Solutol solution was diluted 1:4 (v/v) with sterile 0.9% NaCl to yield a final vehicle composition of 10% DMSO / 10% Solutol / 80% saline and a final drug concentration of 2 mg mL^−1^.

### 4.3 Animal Experiments

NSG mice and C57BL/6 mice were obtained from the Jackson Laboratory and housed under specific‐pathogen‐free conditions in accordance with the institutional guidelines. All procedures were approved by the institutional animal care and use committee of Weill Cornell Medicine.

#### 4.3.1 PDAC Liver Metastasis Model in NSG

Male and female NSG mice (6–7 weeks) received intrasplenic injection of 5 × 10^4^ 8988T/TGL cells in 100 µL PBS to establish liver metastases, as described [62]. For knockdown experiments, 8988T/TGL cells expressing shRenilla or shHRG1#a were pretreated ± doxycycline (0.5 µg mL^−1^, 96 h) and injected intrasplenically; recipients of doxycycline‐pretreated cells were placed on doxycycline chow (LabDiet 5V75; 625 ppm) beginning 1 day prior to injection.

#### 4.3.2 TNBC Lung Metastasis Model in NSG

Female NSG mice (6–7 weeks) received tail vein injection of 5 × 10^4^ MDA‐MB‐231/TGL cells in 100 µL PBS. For inducible knockdown studies, MDA‐MB‐231/TGL cells expressing shRenilla, shHRG1#a, or shHRG1#b were pretreated ± doxycycline (0.5 µg mL^−1^, 96 h) before injection; recipients of doxycycline‐pretreated cells were placed on doxycycline chow (LabDiet 5V75; 625 ppm) beginning 1 day prior to injection.

#### 4.3.3 Murine TNBC Lung Metastasis Model in C57BL/6

Female C57BL/6 mice (6–7 weeks) received tail vein injection of 1 × 10^6^ EO771.lmb cells in 100 µL HBSS.

#### 4.3.4 Healthy Mouse Cohort

Non‐tumor‐bearing male and female C57BL/6 mice received a single intravenous injection of CO‐116 at 0 (untreated), 5, or 15 mg kg^−1^. Blood was collected from the submandibular vein, and tissues were harvested 1 h after the dosing.

#### 4.3.5 Treatment and Imaging

Mice were randomized to the indicated treatment groups according to the schedules specified in the Results and figure legends. BLI was performed using an IVIS Spectrum (PerkinElmer) on Day 0 (post‐injection baseline), Day 1, and weekly thereafter until the experimental endpoint, as described [48, 63]. Body weight was measured weekly. General health and behavior of mice were monitored daily.

### 4.4 Immunoblot Analysis

Cells were treated with 50 µm **CO‐116** or 50 µm **CP‐116** for 5 days with daily medium replacement. After treatment, cells were rinsed once with PBS, scraped into 1 mL PBS, collected by centrifugation, and lysed in RIPA buffer (0.1% SDS, 1% Triton X‐100, 0.5% sodium deoxycholate, 25 mm Tris‐HCl (pH 8.0), 150 mm NaCl, and 1 mm EDTA) supplemented with a protease inhibitor mixture and PhosSTOP (Roche, 4906837001). Proteins were quantified with a Bradford assay (Bio‐Rad). Equal amounts of proteins were separated with SDS‐PAGE and transferred to nitrocellulose membranes. Nitrocellulose membranes were stained with Ponceau S, destained with TBST, incubated in 5% non‐fat milk in TBST for 1 h, probed with primary antibodies against HRG1 (1:1000, St. John's Laboratory, STJ196085), CYP1B1 (1:1,000, Abcam, 32649), SP1 (D4C3) (1:1,000, Cell Signaling, 9389), and β‐actin (1:1,000, Sigma, A2066) overnight, and then were incubated with horseradish peroxidase‐conjugated secondary antibodies. Protein bands were visualized by enhanced chemical luminescence (Cytiva or Pierce).

### 4.5 Tissue Preparation, IHC, and Image Analysis

Mouse tissues were harvested and fixed in 10% buffered formalin overnight at room temperature. Formalin‐fixed/paraffin‐embedded sections (5 µm) were deparaffinized and rehydrated by passage through a graded xylene/ethanol series before staining. Tumor numbers were quantified from one entire H&E‐stained tissue slide of each mouse under the microscope, and tumor sizes were measured from all tumors in one tissue slide of each mouse using the cellSens imaging software (Olympus). IHC was performed using the VECTASTAIN Elite ABC kit with ImmPACT NovaRed Peroxide (Vector Laboratories) following the manufacturer's instructions. The primary antibodies were Ki67 (D3B5) rabbit monoclonal antibody (1:2,000, Cell Signaling Technology, 12202), cleaved caspase‐3 (Asp175) (5A1E) rabbit monoclonal antibody (1:1,000, Cell Signaling Technology, 9664), and SP1 (D4C3) rabbit monoclonal antibody (1:2,000, Cell Signaling Technology, 9389).

Images of Ki67‐stained and cleaved caspase‐3‐stained sections were taken using a 20× objective and imported into QuPath (version 0.3.2). The percentages of Ki67‐positive tumor cells and cleaved caspase‐3‐positive tumor cells within each tumor nodule were quantified. Images of SP1‐stained sections were taken using a 20× objective and imported into HALO software (Indica Labs, version 4.0.5107.407) for analysis. Regions of interest (ROIs) were manually delineated around relevant tumor areas, and the Indica Labs Area Quantification module was used. The software was trained to classify SP1 intensities as weak, moderate, or strong. Additionally, the subcellular localization of staining—hematoxylin‐positive (nuclear) or hematoxylin‐negative (non‐nuclear)—was annotated. All images were batch‐processed using these predefined parameters.

### 4.6 Blood COHb Determination

Terminal blood was drawn 1 h from the submandibular vein and mixed with 1000 IU heparin after the final dose of the prodrugs and kept on ice. COHb was measured with AVoximeter 4000 (Bedford, Massachusetts, USA) according to the manufacturer's manual on the next day. Briefly, 50 µL of blood was added to the cuvette, and the cuvette was inserted into the AVoximeter 4000 to acquire the COHb readings. The instrument was periodically calibrated with the calibration samples purchased from Werfen (Bedford, MA, USA).

### 4.7 Tissue CO Concentration Determination

The amount of CO in the tissue samples was measured by GC according to the reported method [64]. Each tissue was precisely weighed into a 2 mL Eppendorf tube. Ultrapure water was then added to the tissue at a ratio of 4 µL per mg of tissue. The tissues were cut into small pieces using iris scissors while keeping the tube on ice, and then homogenized with Tissue‐Tearor homogenizer (Biospec, Bartlesville, Oklahoma, USA). Subsequently, 300 µL of the tissue homogenate was transferred into 2‐mL headspace vials, followed by the addition of 1200 µL 3.75% 5‐sulfosalicylic acid in ultrapure water in one portion. Each vial was instantly sealed by a crimp seal cap with PTFE‐silicon rubber septa, and then incubated at 37°C for 2 h. Exactly 100 µL of headspace gas from tissue homogenate vial was injected into GC by a gas‐tight syringe with a sample lock valve (Hamilton, Reno, Nevada, USA).

GC was tested on an Agilent 7820a GC system: Purged packed inlet, temperature 150°C. Column: Restek Mole sieve 5A, 80/100 mesh, 0.53 mm × 2 m (Centre County, Pennsylvania, U.S.). Carrier gas: helium. Column flowrate: 4.5 mL/min. Oven temperature: 100°C isocratic for 5 min followed by a bakeout at 300°C for 5 min. Detector: a Restek Methanizer (CH_4_izer, Centre County, Pennsylvania, U.S) is coupled in between the column and the FID detector; it utilizes a nickel catalyst tube and hydrogen gas to convert CO and CO_2_ into methane, thus can be detected with the FID detector; catalyst tube temperature: 380°C; catalyst H_2_ flow rate: 25 mL/min. FID detector: temperature 300°C; H_2_ flow: 15 mL/min; air flow: 400 mL/min.

### 4.8 Measurement of Cell Migration and Intracellular Heme Levels

Transwell migration assays and intracellular heme measurements were performed as previously described [48]. Briefly, cells were subjected to Transwell migration under the indicated treatments, and migrated cells were quantified as reported. Intracellular heme levels were measured using the same heme assay workflow described [48].

### 4.9 Statistical Analysis

Radiance values from bioluminescence imaging and fold change in body weight were natural log‐transformed prior to analysis to improve normality and stabilize variance. Normality assumptions were evaluated using residual diagnostics. No data points were excluded unless pre‐specified experimental criteria were met. Data are presented as mean ± SEM unless otherwise indicated. The sample size (n) for each experiment is indicated in the corresponding figure legends and represents the number of biologically independent animals or samples analyzed. Sample sizes were selected based on prior experience with these metastasis models and the effect sizes observed in preliminary studies and were sufficient (> 80% power) to detect statistically significant differences under the specified models.

For longitudinal radiance and body weight analyses, generalized estimating equations (GEE) were used to account for repeated measurements over time within animals. For comparisons between two groups at a single time point, two‐sided Student's *t*‐tests were performed. For comparisons involving more than two groups, one‐way ANOVA without assuming equal variances was used, followed by appropriate post hoc testing when indicated. Correlation analyses were performed using Pearson's correlation coefficient unless otherwise indicated.

All statistical tests were two‐sided, and a *p‐value* < 0.05 was considered statistically significant. No formal adjustment for multiple testing was applied unless specified. All statistical analyses were performed using SAS version 9.4 (SAS Institute, Cary, NC) and GraphPad Prism (version 10.6.1).

## Author Contributions

T.Z. and Y.‐C.N.D. designed the animal model study and the in vitro study. T.Z., X.C., X.Y., M.G., C.Z., M.Y.Q.C., G.Z., and Q. M. conducted the experiments. T.Z., X.C., X.Y., M.G., D.L., C.Z., M.Y.Q.C., and G.Z. analyzed the data. X.Y., R.K.V., and D.L. synthesized the CO prodrugs and byproducts. Z.C. performed statistical analysis. E.P. contributed to pathological characterization. T.A.I. provided resources. B.W. and C.T. provided additional data interpretation. B.W. supervised the CO prodrugs synthesis and the CO measurement in the blood and tissues. T.Z., X.Y., M.G., and Y.‐C.N.D. wrote materials and methods. Y.‐C.N.D. provided supervision and wrote the manuscript. All authors read the manuscript, agreed with the content, and were given the opportunity to provide input.

## Conflicts of Interest

B.W. is an inventor on patent applications related to metal‐free CO prodrugs filed by GSU. B.W. may receive a share of future licensing revenue. Y.‐C.N.D. is an inventor on a patent application related to methods for treating metastatic cancer using low‐dose CO filed by Cornell University, which is under examination at the United States Patent and Trademark Office. Under Cornell University Policy 1.5, if the invention is licensed, Y.‐C.N.D. may receive a share of future licensing revenue.

## Supporting information




**Supporting File 1**: advs74768‐sup‐0001‐SuppMat.docx.

**Supporting File 2**: advs74768‐sup‐0002‐FigureS1‐S3.pdf.

## Data Availability

The data that support the findings of this study are available in the supplementary material of this article.
