# Supplementary material for: A Metal‐Free Carbon Monoxide Prodrug Suppresses Metastasis of Pancreatic and Breast Cancer
Source: Adv Sci (Weinh). 2026 Mar 20;13(29):e19898. doi: 10.1002/advs.202519898 (PMC13205799; doi:10.1002/advs.202519898)
Supplement: Supplementary file 1 — Supporting File 1: advs74768‐sup‐0001‐SuppMat.docx. [file ADVS-13-e19898-s002.docx]

Supporting Information

Title

**A Metal-Free Carbon Monoxide Prodrug Suppresses Metastasis of Pancreatic and Breast Cancer**

*Tiantian Zhang^1^, Xiang Chen^1^, Xiaoxiao Yang^2^, Muskan Gori^2^, Rohit Kumar Varshnaya^2^, Dongning Liu^2^, Cheryl Zhang^1^, Michelle Yi Qin* *Chen^1^, Zhengming Chen^3^, George Zhang^1^, Erika Patel^1^, Qiyue Mao^2^, Tan A. Ince^1^, Chalet Tan^4^, Binghe Wang^2^, Yi-Chieh Nancy Du^1,*^*

**Supplementary figure legends**

**Figure S1. CO-103 inhibits liver metastasis in an experimental PDAC metastasis model.**
**(A)** Schematic of the experimental design. 8988T/TGL cells (0.05 million) were injected intrasplenically into NSG mice. One day post-injection, mice were randomized into two treatment groups (n = 5 per group): **CO-103** or **CP-103** (control compound) administered IV at 5 mg kg^-1^, three times per week. **(B)** Structures of **CO-103** and **CP-103** and the chemical reaction. **(C)** Quantification of bioluminescence signals over time. Statistical significance was determined using GEE to account for repeated measurements over time. Data are presented as mean ± SEM. *p* =0.0001. **(D)** Representative liver images from each group. **(E)** Representative H&E-stained liver sections from each group. Arrow: metastasis. Scale bar: 200 μm. **(F)** *Ex vivo* BLI data of indicated tissues at the study end point. Data are presented as mean ± SEM. Statistical significance was determined using t-test. Liver: *p* = 0.0336 (*). Pancreas/spleen (Pan/spleen): *p* = 0.0532. Lung/heart: *p* = 0.0648. Kidney: *p* = 0.0161. Femur: *p* = 0.6135. **(G)** Representative *ex vivo* organ BLI images from each group.

**Figure S2. Correlation between migration and intracellular heme levels in MDA-MB-231 and MCF7 cells.**
Scatter plots with linear regression lines and 95% confidence bands show the bivariate relationship between migration and intracellular heme levels in **(A)** MDA-MB-231 cells (*p* = 0.0038) and **(B)** MCF7 cells (*p* <0.0001), using condition means derived from Figure 5B–C. Pearson’s correlation coefficients (r) are reported, with corresponding P values and confidence intervals calculated using Fisher’s Z transformation (biasadj = no).

**Figure S3. Correlation between CO-116 dose and COHb after single-dose administration in healthy C57BL/6 mice.**
Scatter plot with a linear regression line and 95% confidence band showing the bivariate relationship between **CO-116** dose and COHb in healthy, non–tumor-bearing C57BL/6 mice following a single IV injection of **CO-116** (0, 5, or 15 mg kg^-1^; n = 5 per group). Pearson’s correlation coefficient (r) is reported, with the corresponding *p* value (0.0390) and confidence interval calculated using Fisher’s Z transformation (biasadj = no).
