# Supplementary figures and images for: A Metal‐Free Carbon Monoxide Prodrug Suppresses Metastasis of Pancreatic and Breast Cancer
Source: Adv Sci (Weinh). 2026 Mar 20;13(29):e19898. doi: 10.1002/advs.202519898 (PMC13205799; doi:10.1002/advs.202519898)

Figure S1

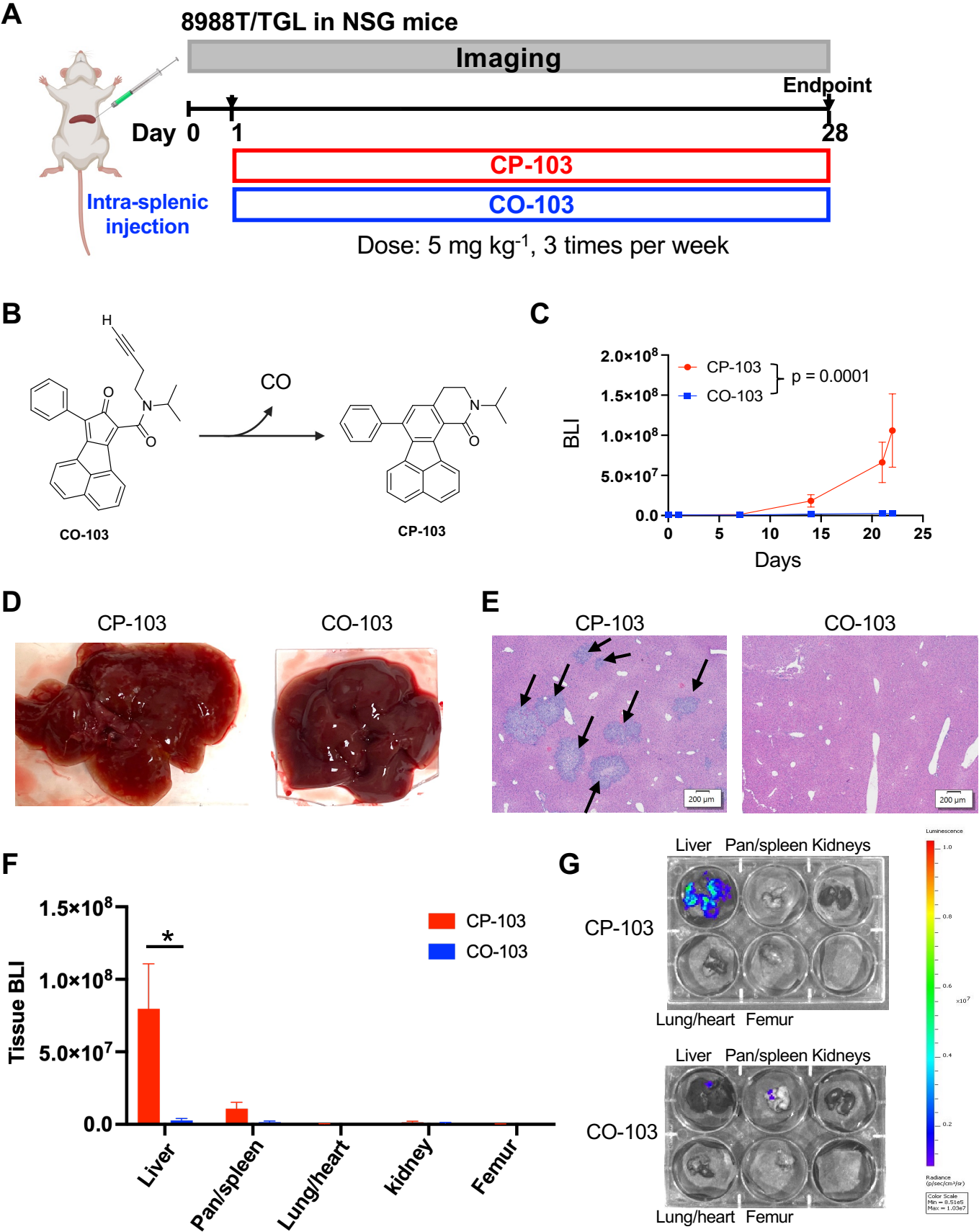

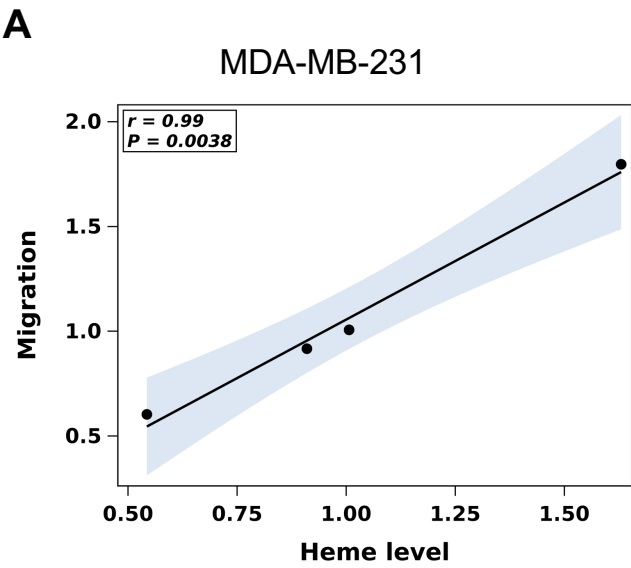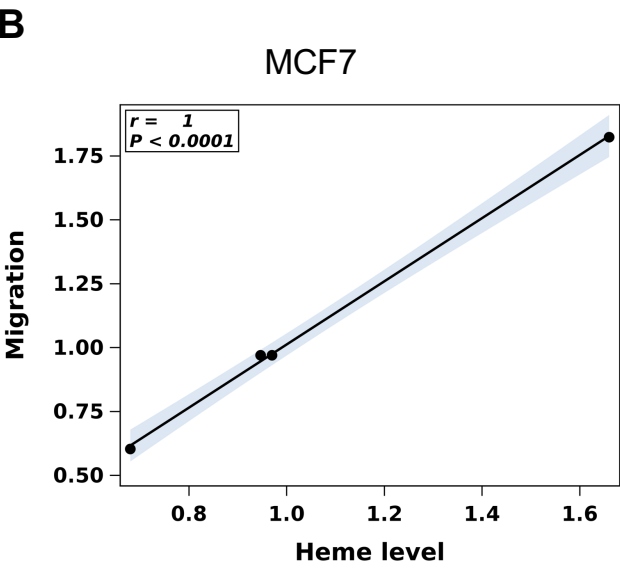

Figure S3

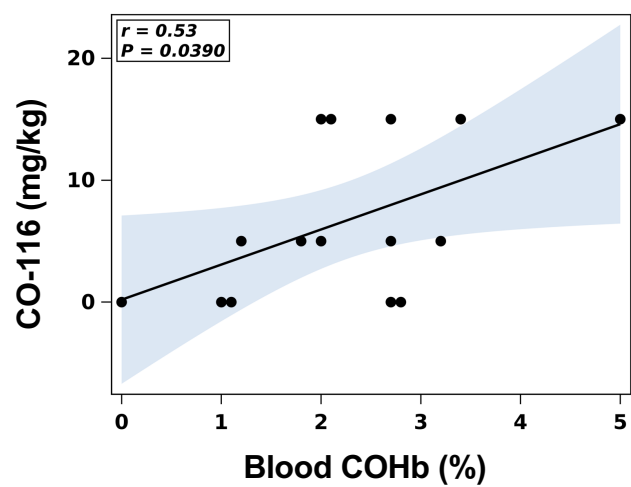

Supplement: Supplementary file 2 — Supporting File 2: advs74768‐sup‐0002‐FigureS1‐S3.pdf. [file ADVS-13-e19898-s001.pdf]
